# Cross-cultural adaptation and validation of the Mental Health Quality of Life (MHQoL) questionnaire in a Chinese-speaking population with chronic musculoskeletal pain

**DOI:** 10.1186/s40359-023-01482-y

**Published:** 2023-12-08

**Authors:** Jialin Wang, Ruirui Wang, Peng Zhao, Tianran Han, Meng Li, Yuwei He, Yan Liu

**Affiliations:** 1 Sports Rehabilitation Research Center, China Institute of Sport Science, Beijing, China; 2 College of Sports Medicine and Physical Therapy, Beijing Sports University, Beijing, China; 3 Centre for Chinese International Education, School of Humanities, Communication University of China, Beijing, China

**Keywords:** Mental Health Quality of Life (MHQoL), Chronic musculoskeletal pain (CMP), Mandarin, translation, Quality of life, Mental health

## Abstract

**Background:**

The Mental Health Quality of Life (MHQoL) questionnaire is concise and suitable for rapid assessment of CMP (chronic musculoskeletal pain) patients in primary care. However, there is a lack of Chinese versions of the MHQoL.

**Objective:**

To cross-culturally translate the MHQoL into Chinese and to assess its psychometric properties in Chinese-speaking patients with CMP.

**Methods:**

The MHQoL was translated into Chinese according to the International Guidelines for the Cross-Cultural Adaptation of Self-Report Measures. 171 CMP patients were recruited to receive the Chinese versions of the MHQoL, SF-36, and HADS tests, and the MHQoL was retested seven days later.

**Result:**

The Chinese version of MHQoL had good retest reliability (MHQoL-7D: ICC = 0.971; MHQoL-VAS: ICC = 0.988) and internal consistency (Cronbach’s alpha = 0.829). It showed a moderate correlation with the SF-36 total score (r=-0.509); the MHQoL-VAS moderately correlated with the Hospital Anxiety Depression Scale (r=-0.548). The MHQoL-7D showed no correlations with the SF-36’s PF (r=-0.083) and BP (r=-0.170), weak correlations with RP (r=-0.284), RE (r=-0.298), and SF (r=-0.380), and moderate-to-strong correlations with GH (r=-0.638), VT (r=-0.480), and MH (r=-0.632).

**Conclusion:**

The Chinese version of the MHQoL can be used in clinical practice and research in Chinese-speaking CMP patients.

## Introduction

Chronic musculoskeletal pain (CMP) occurs in most people at least once in their lifetime [1] and is one of the leading causes of disability in the general population [2]. A survey in the United States showed that one in two adults suffers from CMP, which is more prevalent than cardiovascular disease and chronic respiratory disease combined [3, 4]. As life expectancy increases in society, the health need is no longer merely the absence of disease or infirmity but a complete physical, mental, and social well-being [5]. There is a multidimensional and dynamic integration between these three factors, which influence each other [3]. Studies have shown that reduced quality of life is very common in patients with CMP, which can lead to severe emotional problems in patients with CMP [4, 6, 7]. Many longitudinal observational studies support a strong bidirectional association between mood disorders and chronic pain. Chronic pain greatly increases the risk of mood disorders. Psychological variables such as depression, anxiety, and distress are among the most effective and powerful predictors of the transition from acute to chronic pain [8]. Therefore, quality of life and psychological well-being are important indicators for evaluating the effectiveness of chronic musculoskeletal pain treatment and predictors of the development of chronic musculoskeletal pain.

In recent years, the incidence of CMP has been increasing year by year. In a survey of the chronic pain population in a region of China, the prevalence of chronic pain has increased from 10.8 to 28.7% over the past 15 years, with more than 90% of patients reporting chronic musculoskeletal pain in one or more areas [9]. However, due to the limited resources available to the healthcare system, we need a cost-effective and rapid assessment to accurately determine the quality of life and mental health of patients with CMP [10]. Patient self-reported outcomes (PROMs) have been recognized as highly appropriate assessments and are now widely used to evaluate the quality of primary care [11]. Traditional quality-of-life assessments of patient self-reported outcomes included the EQ-5D or the short form 36 health survey questionnaire (SF-36); however, studies have questioned whether these commonly used tools capture all mental health quality-of-life domains [12–15]. Complex calculations and long measurement times also limit the use of these tools [16]. Therefore, assessment tools with more comprehensive dimensions and shorter measurement times are needed [15].

The Mental Health Quality of Life Questionnaire (MHQoL) is a self-report instrument divided into two subscales, the MHQoL-7D and the MHQoL-VAS, designed to assess dimensions related to the quality of life of people with mental health problems. The instrument has been validated in seven European countries, including the Netherlands. It has good psychometric properties and convergent validity with scales such as the 4-item Patient Health Questionnaire-4 (PHQ-4) and EQ-5D [10, 15]. Therefore, the purpose of this study was to cross-culturally translate the MHQoL into Chinese and to assess its psychometric properties in Chinese-speaking patients with CMP.

## Materials and methods

### Translation and modification procedure

The Chinese version of the scale was cross-culturally translated in six steps following the Self-Report Measurement Cross-Cultural Adaptation Guidelines with the consent of the original authors [17].

#### Stage 1: Initial translation.

Two native Chinese bilingual translators conducted a preliminary translation of the original English version of the MHQoL and obtained two preliminary translations, C1 and C2. The two translators were asked to record the confusing and undecidable parts encountered during the translation process and form a written report. Both bilingual translators have master’s degrees. One has a master’s degree in sports medicine and has studied for two years in an English-speaking country. The other has a Master’s degree in Translation and has studied for five years in an English-speaking country.

#### Stage 2: Synthesis of the first translation version.

The two first-time translators and a note taker synthesize the C1 and C2 versions in a meeting called C3, which requires the two first-time translators to discuss each of the problems they encountered during the translation process and the note taker to record how all the problems were solved and to produce a written report. The C3 version needs to be approved by all the first-time translators and the note taker.

#### Stage 3: Back translation.

Translating the C3 version of the questionnaire back into the original language requires that the original version be completely ignored. This was a validity check to ensure that the translated version reflected the same item content as the original version [17]. The C3 version was translated back into English by two native English speakers with a reasonable level of Chinese language proficiency to form two back-translated versions, H1 and H2, neither of whom had a medical background and any knowledge of the MHQoL scale.

#### Stage 4: Expert committee discussion.

An expert committee consisting of one expert in rehabilitation medicine, one expert in Chinese language and literature, one statistician, and all the translators reviewed the back-translated versions H1 and H2 to check for any meaning bias and formed a written report. In the end, all the translated versions (C1, C2, C3, H1, H2) and written reports were reviewed and the pre-version of the Chinese MHQoL was determined.

#### Stage 5: Chinese MHQoL pre-version testing.

Fifty chronic musculoskeletal pain patients with the corresponding English level were recruited to test the Chinese MHQoL pre-version. All patients were interviewed after completing the questionnaire, and a note-taker recorded their comments or suggestions.

#### Stage 6: The final version of the Chinese MHQoL was finalized by an expert committee.

The pre-version of the Chinese MHQoL was very easy to understand, and there were no sections in which testers expressed doubts or difficulties in understanding. Therefore, the Expert Committee decided that the Chinese version of the MHQoL could be used as the final version.

### Participants and procedures

A total of two groups of participants were recruited. Group A recruited 50 chronic musculoskeletal pain patients who participated in developing the MHQoL questionnaire and completed the SF-36, the hospital anxiety and depression scale (HADS) and the Visual Analogue Scale for pain (VAS for pain). The SF-36 is often used to evaluate the quality of life of CMP patients [18]. Previous studies have confirmed good reliability and validity of the SF-36 in patients with CMP, the baseline SF-36 score is sensitive to changes in pain status and also predicts pain outcomes in patients [19]. Therefore, the SF-36 was used in this study as an external anchor for the validity evaluation of the MHQoL-7D. The HADS has been widely used to assess the psychological status of primary care patients with recognized reliability and validity [20]. Many studies have shown the HADS to be a valid tool for identifying emotional distress in nonpsychiatric patients [21]. Therefore, this study used the HADS as an external anchor to evaluate the validity of the MHQoL-VAS. The VAS for pain has been widely used for pain assessment in patients with CMP with good reliability [22], so this study used the VAS for pain as an external anchor for the evaluation of the total score validity of MHQoL. Two offline tests were conducted by Group A personnel. The first time, a pre-version of MHQoL was filled out. Upon completion, the researcher provided participants with the English version of the MHQoL and asked Group A personnel to provide comments and suggestions on the translation. The SF-36 and HADS scales were also completed. The second completion came seven days later, when the MHQoL questionnaire was completed again by Group A personnel. Since Group A personnel were required to provide comments on the translations, all personnel were required to have English proficiency in College English Test Band 4 (CET-4), which is an English language proficiency test for Chinese students. According to the judgment of the language experts, all the vocabulary in the English version of MHQoL is within the scope of the CET-4.

A complementary group of 121 chronic musculoskeletal pain patients was recruited as Group B and completed the MHQoL questionnaire twice (the second time seven days after the first test), and the first time they also completed the VAS for pain. The study was approved by the Ethical Review Committee of the Institute of Sports Science of the State General Administration of Sport. All participants completed an informed consent form before testing.

All participants were recruited online. We posted participant recruitment information through bulletin boards in surrounding neighborhoods and social media programs with a link to our online questionnaire. Participants were asked to complete the online questionnaire (including demographic information and inclusion and exclusion criteria), which helped the researchers determine whether participants met the inclusion and exclusion criteria, and for those in doubt, the researchers reconfirmed and finalized the participants by phone. A total of two participant recruitment sessions were conducted. Group A was recruited the first time and Group B was recruited two weeks later using the same process. Inclusion criteria: (1) Age > 18 years, able to understand and read Chinese correctly (native Chinese speakers); (2) Persistent or episodic pain in the medial skeleton or peripheral joints lasting more than three months. It includes chronic myofascial pain, fibromyalgia (FM), chronic widespread pain (e.g., chronic fatigue syndrome (CFS)), rheumatoid arthritis, spondyloarthropathies, and a diagnosis of osteoarthritis [23]; (3) A score of ≥ 3 on the Visual Analog Scale (VAS) for pain (4). Participants in Group A are required to have a CET-4 pass. Exclusion criteria were as follows: (1) Patients with acute pain, subacute pain, and chronic non-musculoskeletal pain; (2) Those with mental illnesses such as dementia and cognitive disorders; (3) Those who could not read and correctly understand Chinese.

### Instruments

#### The mental health quality of life questionnaire (MHQoL)

The MHQoL is a self-administered quality-of-life measure developed for evaluating people with subclinical and clinical mental health problems and all mental health services [24]. It consists of two components (the MHQoL-7D and the MHQoL-VAS) and seven questions on seven dimensions (self-image, independence, mood, relationships, daily activities, physical health, and the future), each with four options. In addition, the MHQoL-VAS recorded a respondent’s overall mental health status on a series of levels ranging from 0(“ worst imaginable mental health ”) to 10(“ best imaginable mental health ”) [10, 24]. The total score ranges from 0 to 21, with higher scores indicating better quality of life.

#### Visual analogue scale for pain

The VAS scale for pain consists of a 10-cm measurement line and a pain-related question: What is the average level of pain you have felt in the past 4 weeks? The patient is asked to draw a vertical mark on the 10-cm measuring line, with the leftmost side describing “no pain” and the rightmost side describing “worst pain” [25].

#### 36-item short-form (SF-36)

Quality of life was assessed using the SF-36, which has eight sections that measure eight domains of health-related quality of life (HRQoL): physical functioning (PF), role-physical (RP), or daily role functioning limitations due to physical problems, role-emotional (RE), or daily role functional limitations; body pain (BP); general health perception (GH); vigor (VT); social functioning (SF); and mental health perception (MH). Total scores range from 0 to 100, with higher scores representing better quality of life [26].

#### Hospital anxiety and depression scale (HADS)

This scale is designed to identify depression and anxiety in nonpsychiatric patients and is now widely used in screening to assess the psychological status of patients with musculoskeletal disorders. The 14-item HADS consists of a 7-item anxiety (HADS-A) and a 7-item depression (HADS-D) subscale, with each subscale having a total value ranging from 0 to 21, for a total of 42 points, with the higher the score, the worse the condition. Total HADS values range from 0 (best condition) to 42 (worst condition) [27].

## Statistical procedures

The internal consistency of the MHQoL-7D was calculated using Cronbach’s α, and a value of α > 0.7 indicates good internal consistency. The intraclass correlation coefficient (ICC) was used to calculate retest reliability. The retest reliability of the MHQoL questionnaire was measured for all patients who completed the questionnaire for the first time and seven days later, and an ICC > 0.8 was considered to have a perfect retest reliability. The data distribution was examined using histograms and Q-Q plots, and the Pearson correlation coefficient was used to calculate the aggregation validity if it conformed to a normal distribution; otherwise, the Spearman correlation coefficient was used. The data were tested to be not normally distributed and were calculated using Spearman’s correlation coefficient. Spearman’s correlation coefficient (r) was used to test the correlation between the MHQoL-7D and SF-36 total, between the MHQoL-VAS and HADS, between MHQoL total and VAS for pain to calculate convergent validity. Because the MHQoL-7D dimensions are very well defined, we did not conduct an exploratory factor analysis with each question as a dimension. Instead, we judged construct validity by calculating the correlations between the MHQoL-7D and SF-36 dimensions through the Spearman correlation coefficient. The SF-36 and HADS were chosen because they are currently the most commonly used questionnaires for assessing CMP patients’ quality of life and mental health.

Ceiling and floor effects were assessed by recording the proportion of those with the lowest and highest scores on the MHQOL questionnaire; less than 15% was considered acceptable. Missed or incorrect completion by the participants was recorded. The time spent offline filling out the MHQOL questionnaire by each participant in Group A was recorded.

## Results

### Instruments

171 participants were recruited (50 in Group A and 121 in Group B), and all participants completed all testing processes. A total of 50 were recovered for the SF-36 and HADS scales, and 171 for the MHQoL scale. Demographic and baseline information is shown in Table 1.

**Table 1:** Baseline demographic characteristics of participants with chronic musculoskeletal pain

Characteristics	Number (%) or mean ± SD
**Age (years)**	42 ± 14.88
Range	18–75
**Gender**	
Female	115(67.2%)
Male	56(32.7%)
**Pain location**	
Neck	77(45.0%)
Shoulder	21(12.2%)
Arm	6(3.5%)
Wrist	3(1.7%)
Thoracic vertebra	2(1.1%)
Low-back	28(16.3%)
Knee	21(12.2%)
Leg	5(2.9%)
Ankle	3(1.7%)
Others	5(2.9%)
**Education** ^**#**^	
High education	91(53.2%)^*^
Middle education	63(36.8%)
Low education	17(9.9%)
**SF-36**	66.29 ± 12.91
**HADS**	9.36 ± 5.41
**MHQoL-7D**	12.89 ± 2.83
**VAS-10 cm**	4.90 ± 1.45

SD: standard deviation; SF-36: 36-item Short-Form; HADS: Hospital Anxiety and Depression Scale; MHQoL: Mental Health Quality of Life questionnaire. ^#^ Referring to ISCED 2011, Low education refers to early childhood education, primary education, lower secondary education. Middle education refers to upper secondary education, post-secondary non-tertiary education. High education refers to short-cycle tertiary education, bachelor or equivalent, master or equivalent, doctoral or equivalent [28]. ^*^All persons in group A belonged to higher education. 41 persons in group B belonged to higher education, which is 34% of the total number of persons in group B

### Internal consistency

The Cronbach’s alpha for the MHQoL-7D was 0.829, indicating that the measure is highly reliable. Item analysis showed that all items were moderately correlated with the MHQoL-7D total score, ranging from 0.602 to 0.774. When any item was deleted, Cronbach’s alpha was greater than 0.7 and remained stable. The internal consistency of each item is shown in Table 2.

**Table 2:** Test-retest reliability and internal consistency (N = 171)

Item		ICC (95%CI)	Corrected item-total correlation	Cronbach’s α if the item was deleted
**MHQoL-7D**				
1-Self-image		0.960(0.946 ~ 0.971, *P* < 0.01)	0.669	0.813
2-Independence		0.948(0.929 ~ 0.962, *P* < 0.01)	0.702	0.810
3-Mood		0.961(0.947 ~ 0.971, *P* < 0.01)	0.716	0.804
4-Relationships		0.940(0.919 ~ 0.956, *P* < 0.01)	0.690	0.808
5-Daily activities		0.923(0.897 ~ 0.943, *P* < 0.01)	0.774	0.791
6-Physical health		0.872(0.829 ~ 0.904, *P* < 0.01)	0.602	0.820
7-Future		0.949(0.931 ~ 0.962, *P* < 0.01)	0.762	0.793
Total		0.971(0.961 ~ 0.979, *P* < 0.01)	—	—
**MHQoL-VAS**		0.988(0.984 ~ 0.991, *P* < 0.01)	—	—

ICC: intraclass correlation coefficient; CI: confidence interval; MHQoL: Mental Health Quality of Life Questionnaire; VAS: Visual Analog Scale

### Test-retest reliability

The retest reliability of the Chinese version of the MHQoL, ICC = 0.971 (0.961 ~ 0.979, *p* < 0.01) for the quality-of-life component (MHQoL-7D) and ICC = 0.988 (0.984 ~ 0.991, *p* < 0.01) for the mental health component (MHQoL-VAS), suggests that the MHQoL across all test times and occasions is highly stable. The retest reliability scores for each item are shown in Table 2.

### Validity

The correlation between the MHQoL-7D (quality of life component) and the SF-36 total score was − 0.509, considered a moderate correlation. The correlation between the MHQoL-VAS and the Hospital Anxiety Depression Scale was − 0.548, which was considered a moderate correlation. The MHQoL-7D was uncorrelated with PF and BP, weakly correlated with RP, RE, and SF, and moderately to strongly correlated with GH, VT, and MH compared to the SF-36 subfields. The correlation between the MHQoL total score and VAS-10 cm was − 0.441, a moderate correlation. The data can be viewed in Table 3.

**Table 3:** Validity of the MHQoL.

MHQoL-7D (N = 50)	Spearman’s correlation coefficient (r)	MHQoL-VAS (N = 50)	Spearman’s correlation coefficient (r)	MHQoL-Total (N = 171)	Spearman’s correlation coefficient (r)
**SF-36 (Total)**	-0.509	**HADS**	-0.548	**VAS-10 cm**	-0.441
**PF**	-0.083*				
**RP**	-0.284				
**BP**	-0.170*				
**GH**	-0.638				
**VT**	-0.480				
**SF**	-0.380				
**RE**	-0.298				
**MH**	-0.632				

*Insignificant; SF-36: 36-item Short-Form; MHQoL: Mental Health Quality of Life Questionnaire; VAS: Visual Analog Scale

### Ceiling and floor effects and acceptability

Of all 171 participants, the lowest score for MHQoL-7D was 7 (9 persons, 5.2%) and the highest score was 21 (1 person, 0.5%), the lowest score for MHQoL-VAS was 1, (1 person, 0.5%) and the highest score was 10 (19 persons, 11.1%), no significant ceiling and floor effect was observed.

All patients completed all items of the MHQoL scale without errors or omissions. The mean time to complete the MHQoL scale for the 50 patients in group A was 51.41 ± 18.30s.

## Discussion

To the best of our knowledge, this study is the first in which the MHQoL scale was cross-culturally translated and adapted in Chinese-speaking CMP patients. The Chinese version of the MHQoL showed good internal consistency and retest reliability with good convergent validity in CMP patients. In addition, no ceiling or floor effects were found for the Chinese version of the MHQoL. The questionnaire was easily accessible to patients and took less than one minute to complete.

The Chinese version of the MHQoL has good internal consistency, with a Cronbach alpha value of 0.829. This result is similar to the English version validated in seven European countries. The overall result for the seven European countries was 0.82 (Germany 0.81, UK 0.87, Denmark 0.82, Netherlands 0.84, France 0.80, Portugal 0.78, Italy 0.83) [24]. The item-total correlations (Pearson coefficients ranging from 0.602 to 0.774) for the corrected 11 items were above the minimum recommended level of 0.2. Cronbach’s alpha was greater than 0.7 and remained stable when any of the items were removed. The indicates that each item assessed the same concept.

The interval between the first and second tests was seven days. This interval is suitable for assessing the reliability of retesting because it prevents the patient from recalling the first test and ensures that the patient’s condition has not changed [29]. Both the MHQoL-7D total score (ICC = 0.971) and the MHQoL-VAS (ICC = 0.988) showed good retest reliability, and the individual items of the MHQoL-7D also had very high retest reliability (0.872 to 0.961), which is consistent with previously published studies [10].

Construct validity was judged by measuring the correlations between the MHQoL-7D and the SF-36 dimensions, with the resulting results indicating whether the MHQoL-7D correlated with these constructs. We did not conduct an exploratory factor analysis of the MHQoL because the dimensions of the MHQoL were sufficiently explicit that each question was a dimension [24]. Construct validity was judged by measuring the correlations between the MHQoL-7D and the SF-36 dimensions, with the resulting results indicating whether the MHQoL-7D correlated with these constructs. There were no correlations with the SF-36’s PF and BP, weak correlations with RP, RE, and SF, and moderate-to-strong correlations with GH, VT, and MH. This result is related to the different measurement dimensions of the MHQoL-7D and the SF-36 [26]. The two uncorrelated components, PF and BP, are usually assessed using specialized scales. A systematic review of chronic musculoskeletal pain showed that 97% of studies assessed pain intensity, and 87% assessed physical function [30]. The absence of these two components does not affect the assessment and saves time. The reason for not reaching a strong correlation between the MHQoL-VAS and the HADS may be that the HADS scale evaluates only two parts of the psychological condition, depression, and anxiety, which, although the most common mental health problem, is still not sufficiently comprehensive, and the MHQoL-VAS assesses the overall mental health condition.

The completion rate of the Chinese version of the MHQoL was 100%, with no floor or ceiling effect. The presence of a floor or ceiling effect may be due to a lack of extreme items at the low or high end of the scale, indicating that the scale has limited content validity to differentiate between patients with the lowest or highest scores [18]. All patients in this study had no difficulty completing the questionnaire, suggesting that the questionnaire was culturally adapted and acceptable.

There are also some limitations of this study. Group B in our study was administered the questionnaire via the Internet, and the inability to monitor the status of patient completion may have impacted the quality of the results. The types of musculoskeletal pain in our included patients were not sufficiently averaged. Finally, the responsiveness of the Chinese version of the MHQoL was not measured in this study; therefore, the extent to which the MHQoL can detect changes in severity in patients with chronic musculoskeletal pain is currently unknown, and future longitudinal studies will be needed to test this result.

## Conclusion

The Chinese version of the MHQoL was standardized and cross-culturally translated and had good internal consistency and retest reliability in Chinese-speaking patients with chronic musculoskeletal pain, acceptable structural validity, convergent validity, and good acceptability, with no ceiling effect or floor effect observed. This study demonstrates that the Chinese version of the MHQoL can be used in clinical practice and research in Chinese-speaking chronic musculoskeletal patients.

## Data Availability

The datasets used and/or analyzed during the current study are available from the corresponding author on reasonable request.

## Abbreviations

CMP: Chronic musculoskeletal pain
PROMs: Patient self-reported outcomes
SF-36: Short form 36 health survey questionnaire
MHQoL: The Mental Health Quality of Life Questionnaire
PHQ-4: 4-item Patient Health Questionnaire-4
HADS: Hospital anxiety and depression scale
FM: Fibromyalgia
CFS: Chronic fatigue syndrome
VAS: Visual Analog Scale
HRQoL: Health-related quality of life
PF: Physical functioning
RP: Role-physical
RE: Role-emotional
BP: Body pain
GH: General health perception
VT: Vigor
SF: Social functioning
MH: Mental health perception
ICC: Intraclass correlation coefficient
